# Safety and efficacy of trofinetide in Rett syndrome: a systematic review and meta-analysis of randomized controlled trials

**DOI:** 10.1186/s12887-024-04526-3

**Published:** 2024-03-23

**Authors:** Abdallah Abbas, Aya M Fayoud, Mostafa Hossam El Din Moawad, Abdullah Ashraf Hamad, Heba Hamouda, Eman A. Fouad

**Affiliations:** 1 Faculty of Medicine, Al-Azhar University, Damietta, Egypt; 2 Faculty of Pharmacy, Kafr El sheik university, Kafr El Sheik, Egypt; 3 Faculty of Pharmacy Clinical Department, Alexandria University, Alexandria, Egypt; 4 Faculty of Medicine, Suez Canal University, Ismailia, Egypt; 5 Faculty of Medicine, Menoufia University, Menoufia, Egypt; 6 Department of Pediatrics, Ubbo-Emmius-Klinik, Aurich, Germany

**Keywords:** Daybue, Neurodevelopmental disorder, Rett syndrome, Trofinetide

## Abstract

**Introduction:**

Rett syndrome is a rare genetic neurodevelopmental disorder that predominantly impacts females. It presents with loss of acquired skills, impaired communication, and stereotypic hand movements. Given the limited treatment options for Rett syndrome, there is a dire need for effective interventions.

**Objective:**

To evaluate the safety and efficacy of trofinetide in Randomized Controlled Trials (RCTs) that report on Rett syndrome patients.

**Methods:**

We identified 109 articles from four databases (Scopus, PubMed, Web of Science, and Cochrane CENTRAL). After removing the duplicates, we narrowed them down to 59 articles for further assessment. We included RCTs that evaluated the efficacy and safety of trofinetide in patients with Rett syndrome. Three studies were eligible for inclusion. Two independent reviewers evaluated the identified studies’ titles, abstracts, and full texts, extracting pertinent data. We assessed the quality of the studies using the Cochrane Risk of Bias (RoB) 2.0 tool. We then conducted a meta-analysis using the fixed effects model in the case of insignificant heterogeneity; otherwise, we used the random effects model. Based on the nature of the outcome, we analyzed the mean difference or the odds ratio. Analysis was conducted using RevMan version 5.3.

**Results:**

Among the analyzed outcomes in 181 patients in the trofinetide group and 134 patients in the placebo group, significant improvement in Rett Syndrome Behavior Questionnaire (RSBQ) scores was observed at 200 mg dosage (overall mean difference: -3.53, *p* = 0.001). Clinical Global Impression-Improvement (CGI-I) scores improved considerably at 200 mg dosage (overall mean difference: -0.34, *p* < 0.0001). No substantial changes were observed in Motor Behavioral Assessment (MBA) or Top 3 Caregiver Concerns. We evaluated Treatment Emergent Adverse Events (TEAEs) across the various dosages and noted significant associations with diarrhea (200 mg), vomiting (200 mg), and irritability (200 mg). However, we did not find a significant association between any of the dosages and the incidence of decreased appetite.

**Conclusion:**

Trofinetide demonstrated potential in improving RSBQ and CGI-I scores at 200 mg dosage. Although no substantial changes were found in MBA and top 3 caregiver concerns. Adverse events were linked to specific dosages.

**Supplementary Information:**

The online version contains supplementary material available at 10.1186/s12887-024-04526-3.

## Introduction

Rett syndrome is a genetic neurodevelopmental disorder that predominantly affects females, with a prevalence ranging from 1:10,000 to 1:23,000 female live births [1]. A recent systematic review and meta-analysis by Petriti et al. (2023) reported a pooled prevalence estimate of 7.1 cases per 100,000 females and a prevalence range of approximately 5 to 10 cases per 100,000 females [2].

Rett syndrome was previously classified as one of the Autism Spectrum Disorders (ASD) in the fourth edition of the Diagnostic and Statistical Manual of Mental Disorders (DSM). However, it is no longer included as an ASD in the fifth edition of DSM [3].

This syndrome is caused by mutations in the MECP2 gene, ultimately leading to cognitive impairment, communication dysfunction, stereotypic movement disorder, and growth failure [4].

Rett syndrome presents a significant clinical burden [4], given the limited treatment options and the unmet need for effective interventions [4]. Trofinetide is a synthetic analog of glycine-proline-glutamate, a naturally occurring tripeptide cleaved from insulin-like growth factor 1 (IGF-1) [5]. It has shown promise as a potential therapeutic agent for Rett syndrome [5].

While the exact mechanism of action of trofinetide remains unclear, it is thought to enhance neuronal morphology and synaptic functioning [5, 6]. Studies have demonstrated that trofinetide restores synaptic structure, mitigates the impact of neuro-inflammatory substances in the brain, boosts antioxidant responses, reduces injury-induced apoptosis, normalizes the synthesis of essential proteins, reinstates brain homeostasis, and increases the concentration of IGF-1 in the Central Nervous System (CNS) [5, 6].

Previous trials have investigated the efficacy and safety of trofinetide in Rett syndrome. In a phase II trial, trofinetide demonstrated clinical benefits over placebo in clinician- and caregiver-assessed efficacy outcomes [6]. Trofinetide has also been shown to significantly improve the Rett Syndrome Behavior Questionnaire (RSBQ) total score in females with Rett syndrome, when compared to placebo. In addition, changes from baseline in all RSBQ subscores were directionally in favor of trofinetide [7]. However, it is important to note that in addition to the limitation in available evidence, conflicting findings do exist. The mechanism of action of trofinetide and its precise effects in Rett syndrome have still not been well established [5, 6]. Furthermore, there continues to be a gap in the literature with regards to clinical practice guidelines and treatment pattern data [4, 8]. The aim of this systematic review and meta-analysis is to evaluate and summarize the findings of relevant Randomized Controlled Trials (RCTs) that report on the safety and efficacy of trofinetide in Rett syndrome.

## Methods

All steps in this study were performed in strict accordance with the Cochrane Handbook for Systematic Reviews of Interventions [9]. Through out this systematic review and meta-analysis, our reporting followed the Preferred Reporting Items for Systematic reviews and Meta-Analyses (PRISMA) statement guidelines [10].

### Search strategy and eligibility criteria

#### Search strategy

PubMed, Scopus, Cochrane CENTRAL, and Web of Science (WoS) were searched from inception until July 27, 2023, using the following query: ((trofinetide) AND (“Rett syndrome” OR “Rett Disorder” OR “Rett’s Disorder” OR “Rett’s Syndrome” OR “Retts Syndrome” OR “Cerebroatrophic Hyperammonemia*” OR “Autism Dementia Ataxia Loss of Purposeful Hand Use Syndrome”)). Two investigators independently checked this process (A.A.H, H.M). Conflicts were settled through discussions, consensus, and input from a third author, if necessary.

#### Eligibility criteria

We included RCTs that reported the safety and efficacy of trofinetide on Rett syndrome patients. We excluded observational studies, non-randomized trials, and studies from which data could not be reliably extracted. Eligibility screening was conducted in two stages, each by two independent reviewers: (a) title and abstract screening for studies matching the pre-determined inclusion criteria, and (b) full-text screening for studies eligibile for quantitative analysis. Conflicts were settled through discussions, consensus, and input from a third author, if necessary.

### Data extraction

Two authors extracted the relevant data. The extracted data included the following: (a) study characteristics, (b) participant characteristics, (c) risk of bias domains, and (d) study outcomes, including efficacy outcomes (RSBQ, Clinical Global Impression-Improvement (CGI-I), top 3 caregiver concerns, and Motor Behavioral Assessment (MBA) change index) and safety outcomes (diarrhea, vomiting, pyrexia, irritability, and decreased appetite). These safety outcomes were the most frequently occurring Treatment Emergent Adverse Events (TEAEs) in the eligible studies.

### Risk of bias assessment

Two separate reviewers utilized the Cochrane Risk of Bias (RoB) 2.0 assessment tool to evaluate the quality of the included studies as outlined in Chapter 8.5 of the Cochrane Handbook for Systematic Reviews of Interventions 5.1.0 [9]. This tool can assess five types of bias: selection, performance, detection, attrition, and reporting. The authors evaluated each of the studies to determine if they possess a low, high, or uncertain risk of bias in each domain.

### Statistical analysis

Data for dichotomous outcomes were extracted and pooled as an Odds Ratio (OR) with a 95% confidence interval in a fixed effects model. We used RevMan version 5.3 for Windows [11] to conduct the meta-analysis and Meta Converter tool to calculate the change from the baseline [12]. The Chi-square test was used to assess heterogeneity, and the I-square test was used to measure its extent. If significant heterogeneity was found (Chi-square *p* < 0.1), the analysis was conducted using the random effects model, and sensitivity analysis was performed to resolve the heterogeneity.

A subgroup analysis by trofinetide doses was performed to precisely evaluate the effect of the various doses on safety and efficacy outcomes. We also conducted sensitivity analysis to confirm the robustness of our findings.

## Results

### Search strategy and screening

**Fig. 1:**
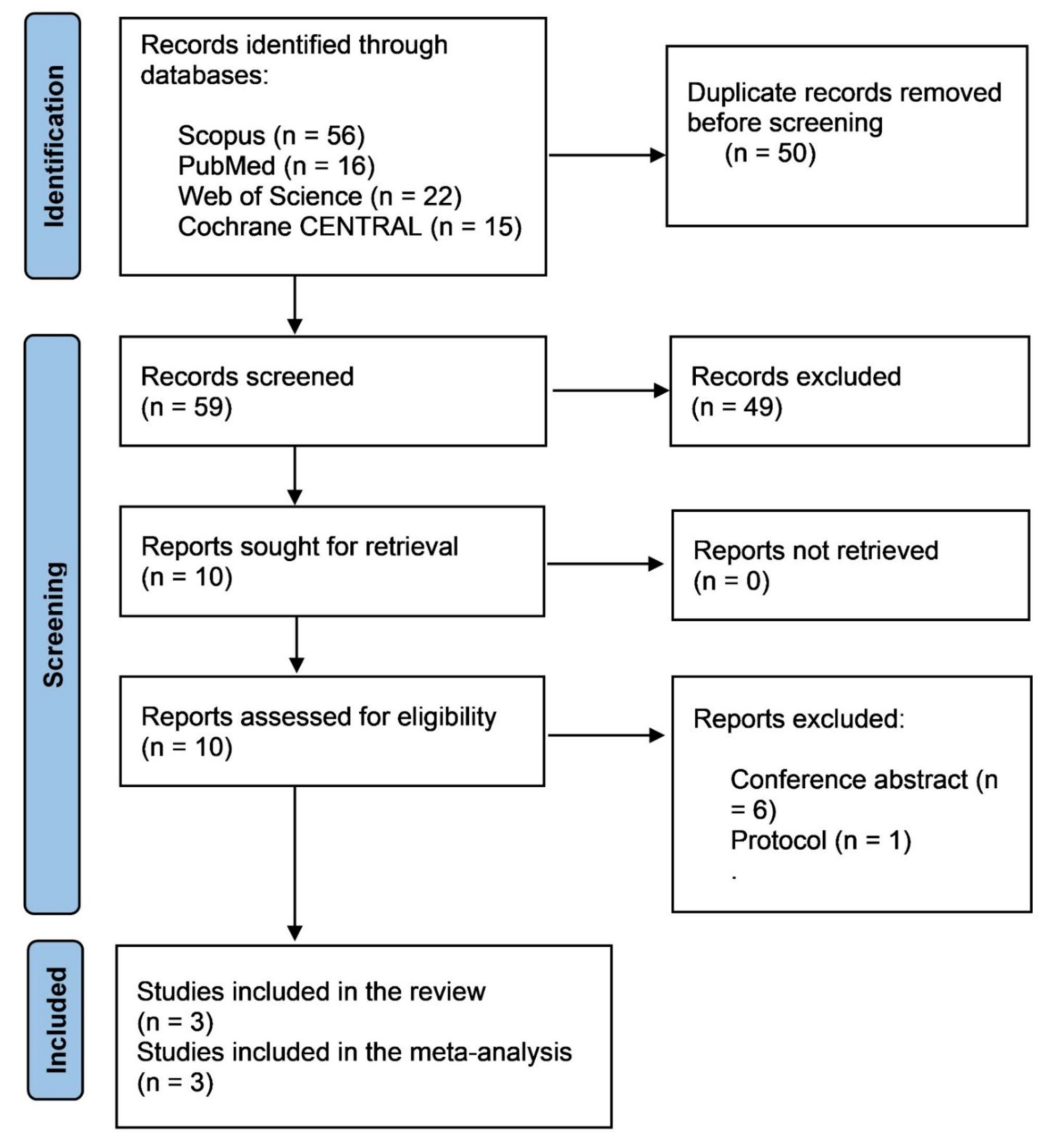
PRISMA flow diagram showing our search and screening process

The databases we used to conduct our research yielded 109 articles to review. A total of 59 studies were left after duplicates were eliminated for assessment. Three studies [6, 13, 14] That met our criteria and qualified for the systematic review and meta-analysis were included after looking at the remaining ten full texts, as shown in (Fig. 1).

### Baseline characteristics

The three studies included in our meta-analysis had 181 patients in the trofinetide group receiving various doses and 134 patients in the placebo group, as illustrated in Tables 1 and 2.

**Table 1:** Shows the summary of the included studies

Study ID	NCT Number	Study Design	Setting	Participants (Inclusion Criteria)	Intervention	Comparison	Dose of Intervention	Conclusion	Follow-up
Glaze 2017		Multicenter randomized controlled trial	USA	Adolescent and adult females with Rett syndrome	Trofinetide	Placebo orally twice a day for 28 days	35 mg/kg orally twice a day for 28 days or 70 mg/kg orally twice a day for 28 days	Trofinetide at a high dose (70 mg/kg) was more effective than placebo in Rett syndrome	40 weeks
Glaze 2019	NCT02715115	Multicenter double-blind placebo-controlled trial	USA	Adolescent or children females with Rett syndrome	Trofinetide	Placebo orally or via a gastrostomy tube twice daily for 42 days	50 mg/kg, 100 mg/kg, or 200 mg/kg orally or via gastrostomy tube twice daily for 42 days	Trofinetide at a dose of 200 mg/kg bid was tolerable and effective in children with Rett syndrome	66 days
Neul 2023	NCT04181723	A randomized, parallel-group, placebo-controlled study	USA	Girls and women 5–20 years of age with Rett syndrome	Trofinetide	Placebo orally twice daily for 12 weeks	200 mg orally twice daily for 12 weeks	Trofinetide was superior to placebo in improving caregiver (RSBQ) and clinician (CGI-I) outcomes	30 days

**Table 2:** Shows the baseline characteristics of the included studies

Study ID	Glaze et al., 2017			Glaze et al., 2019				Neul et al., 2023	
	Trofinetide		Placebo	Trofinetide			Placebo	Trofinetide	Placebo
	35 mg/kg	70 mg/kg		50 mg/kg	100 mg/kg	200 mg/kg		200 mg/kg	
Number of patients (n)	13	17	Doses: (35 mg/kg) = 5, (70 mg/kg) = 11	15	16	27	24	93	94
Gender [Female n (%)]	30 (100%)		16 (100%)	58 (100%)			24 (100%)	93 (100%)	94 (100%)
Age, year [Mean (SD)]	22.62 (5.582)	24.52 (5.853)	Doses: (35 mg/kg) = 32.09 (9.324), (70 mg/kg) = 27.09 (8.357)	10.06 (3.18)	10.81 (3.10)	9.23 (3.88)	9.38 (3.26)	11.0 (4.69)	10.9 (4.57)
BMI, kg/cm2 [Mean (SD)]	25.06 (7.930)	20.48 (6.765)	Doses: (35 mg/kg) = 24.66 (8.04), (70 mg/kg) = 19.24 (3.598)	16.50 (3.61)	17.70 (5.06)	16.31 (3.57)	16.00 (2.85)		
**Ethnicity, n (%)**									
Hispanic	0	2 (12%)	Doses: (35 mg/kg) = 1 (20%), (70 mg/kg) = 0	1 (7%)	1 (6%)	6 (22%)	0		
Not Hispanic	13 (100%)	15 (88%)	Doses: (35 mg/kg) = 4 (80%), (70 mg/kg) = 11 (100%)	14 (93%)	14 (88%)	21 (78%)	24 (100%)		
**Race, n (%)**									
Asian	0	1 (6%)	Doses: (35 mg/kg) = 0, (70 mg/kg) = 0	0	0	2 (7%)	1 (4%)	5 (5.4%)	1 (1.1%)
Black or African-American	3 (23%)	1 (6%)	Doses: (35 mg/kg) = 0, (70 mg/kg) = 0	0	1 (6%)	0	0	1 (1.1%)	1 (1.1%)
White	10 (77%)	15 (88%)	Doses: (35 mg/kg) = 5 (100%), (70 mg/kg) = 11 (100%)	15 (100%)	15 (94%)	25 (93%)	22 (92%)	82 (88.2%)	90 (95.7%)
Other	0			0	0	0	1 (4%)	4 (4.3%)	2 (2.1%)

### Quality assessment

Three studies were assessed using the RoB 2.0 tool; one study was found to have an overall score of “some concerns” and two studies were found to be of “low risk” (Fig. S1).

### Statistical analysis

We analyzed nine outcomes to assess the safety and efficacy of trofinetide in Rett syndrome. We used a fixed effects model across all the outcomes; no statistically significant heterogeneity was found in any of the nine outcomes analyzed.

#### Analysis at 200 mg dosage

##### RSBQ scores

**Fig. 2:**
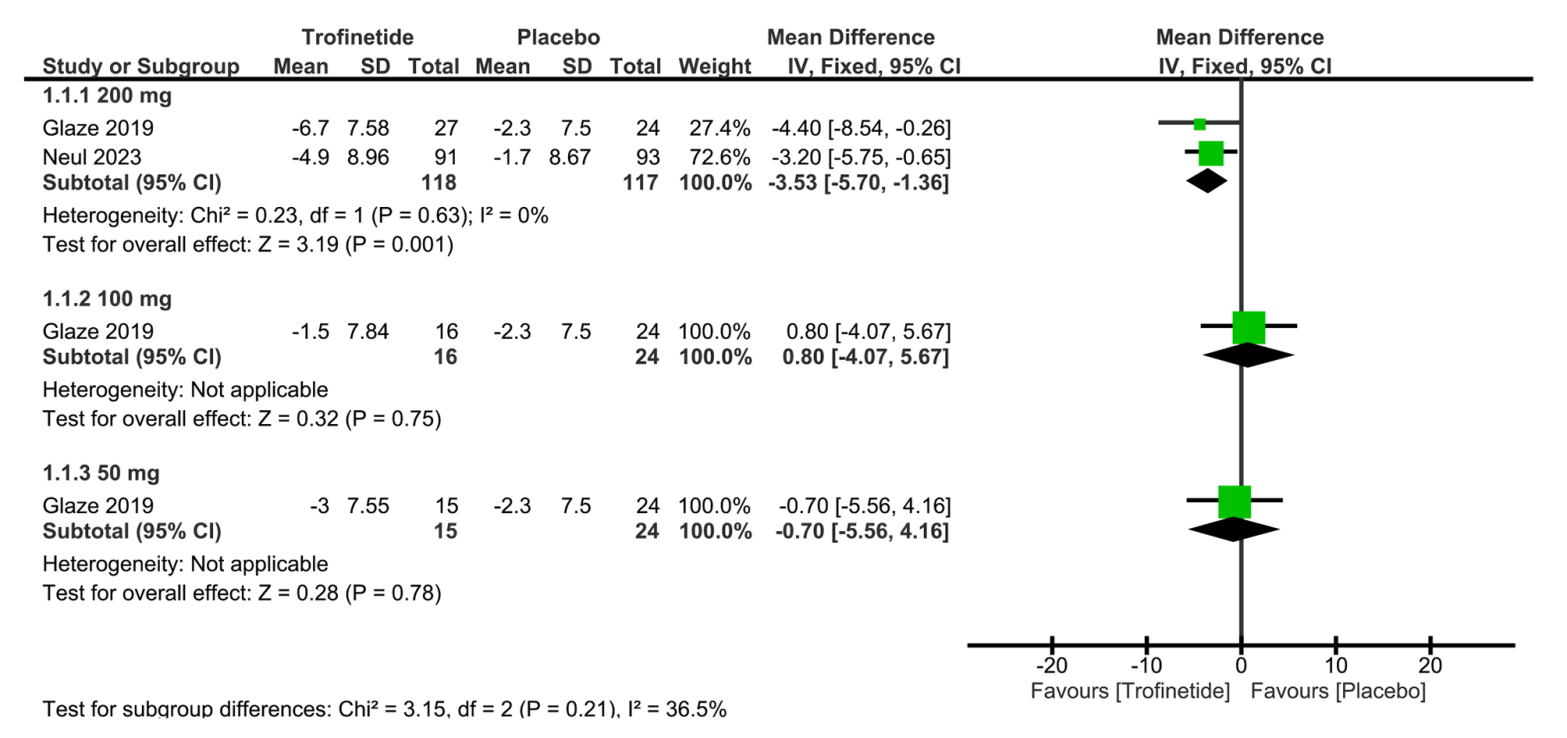
Shows the analysis of RSBQ scores between trofinetide and placebo groups at various dosages

Our analysis of two studies [6, 13] involving trofinetide at the 200 mg dosage revealed an overall mean difference in RSBQ scores of -3.53 (95% CI: -5.70, -1.36, *p* = 0.001), indicating a statistically significant discrepancy between the trofinetide group and the placebo group, favoring trofinetide (Fig. 2).

##### CGI-I scores

**Fig. 3:**
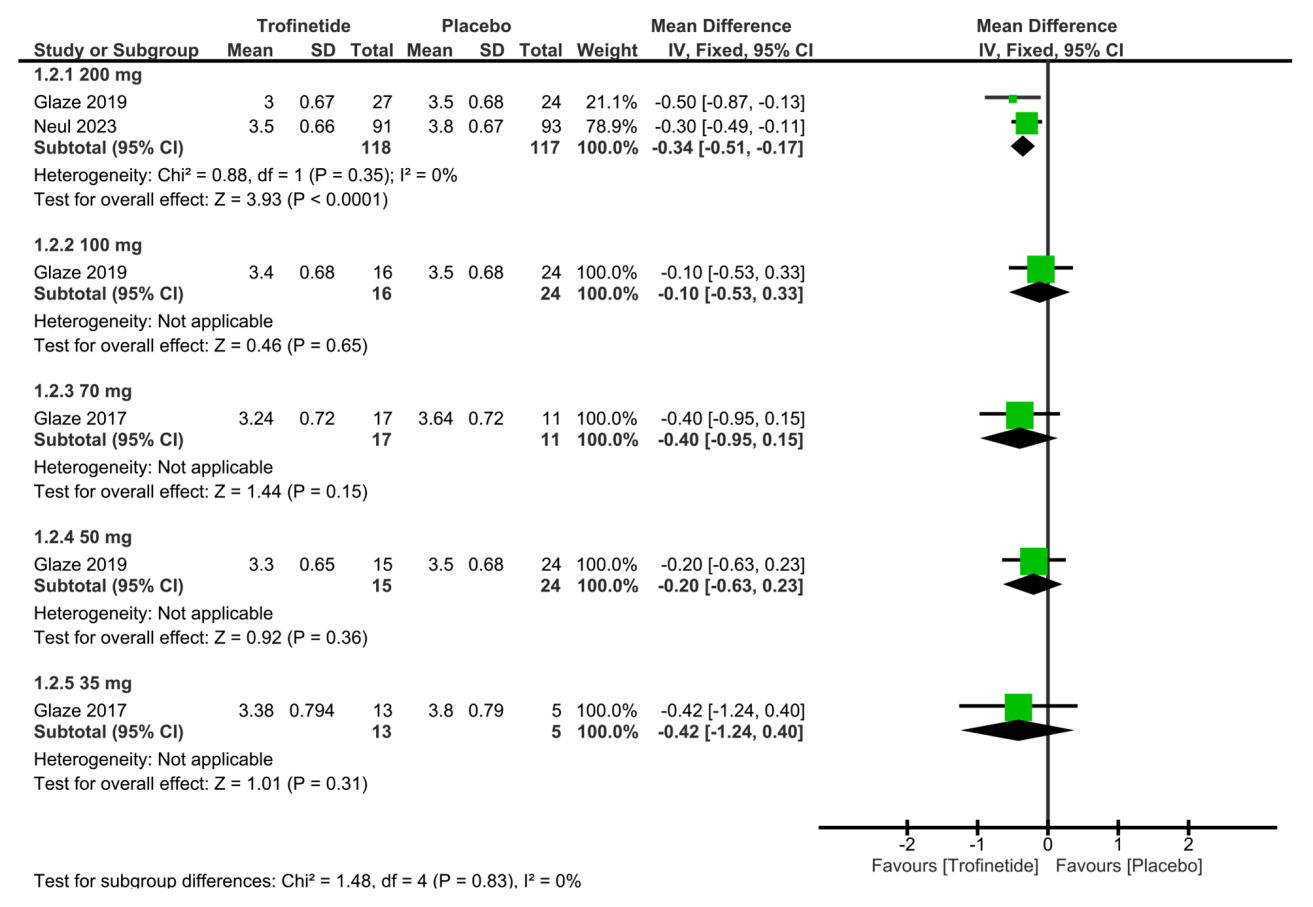
Shows the analysis of CGI-I scores between trofinetide and placebo groups at various dosages

After analyzing data from two studies involving trofinetide at a dosage of 200 mg [6, 13]. We found the overall mean difference in CGI-I scores to be -0.34 (95% CI: -0.51, -0.17, *p* < 0.0001), suggesting a statistically significant difference between the trofinetide group and the placebo group, favoring trofinetide (Fig. 3).

##### MBA scores

**Fig. 4:**
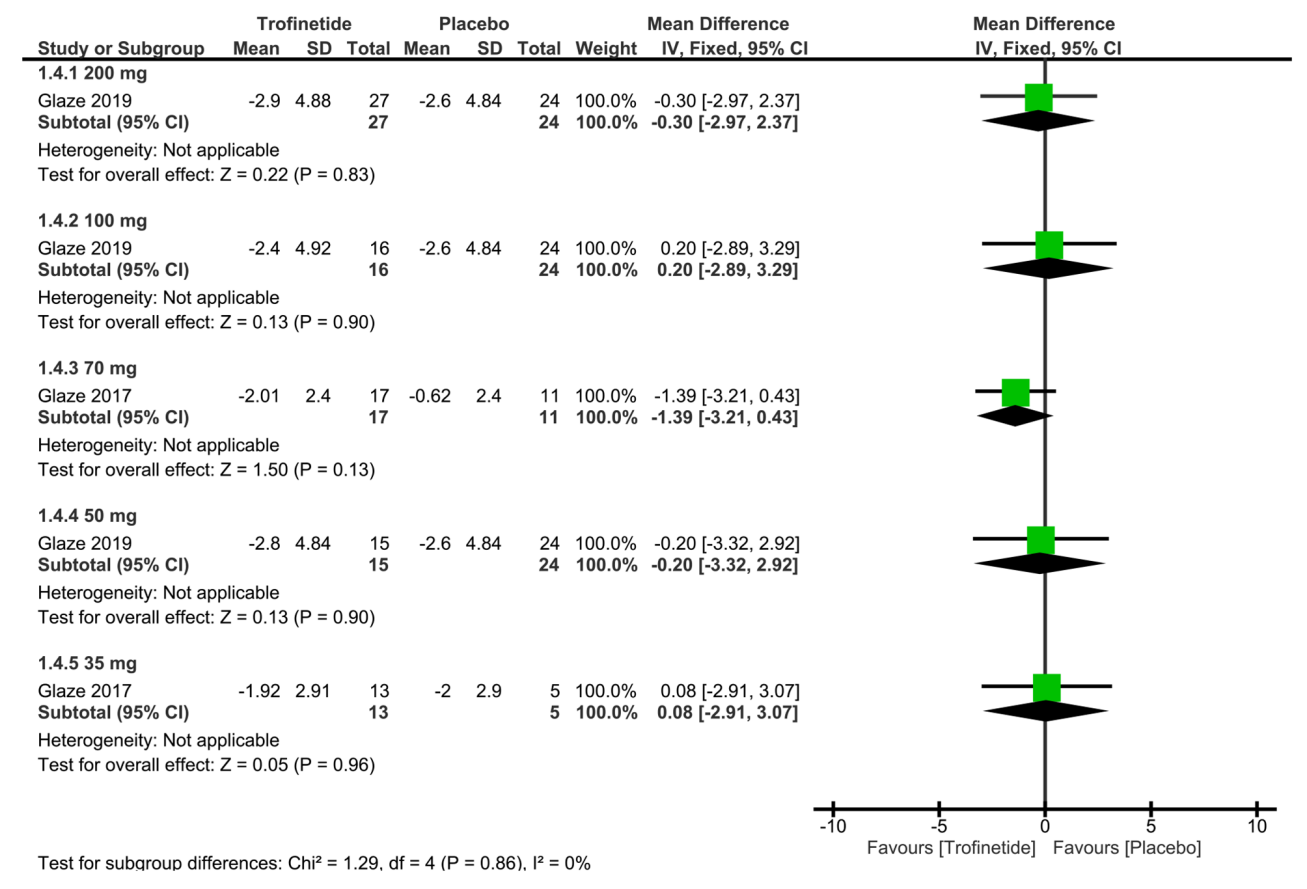
Shows the analysis of MBA scores between trofinetide and placebog groups at various dosages

One study provided data on MBA scores at the 200 mg dosage [13]. The overall mean difference in MBA surfaced as -0.30 (95% CI: -2.97, 2.37, *p* = 0.83), showing an insignificant difference between the two groups (Fig. 4).

##### Top 3 caregiver concerns

**Fig. 5:**
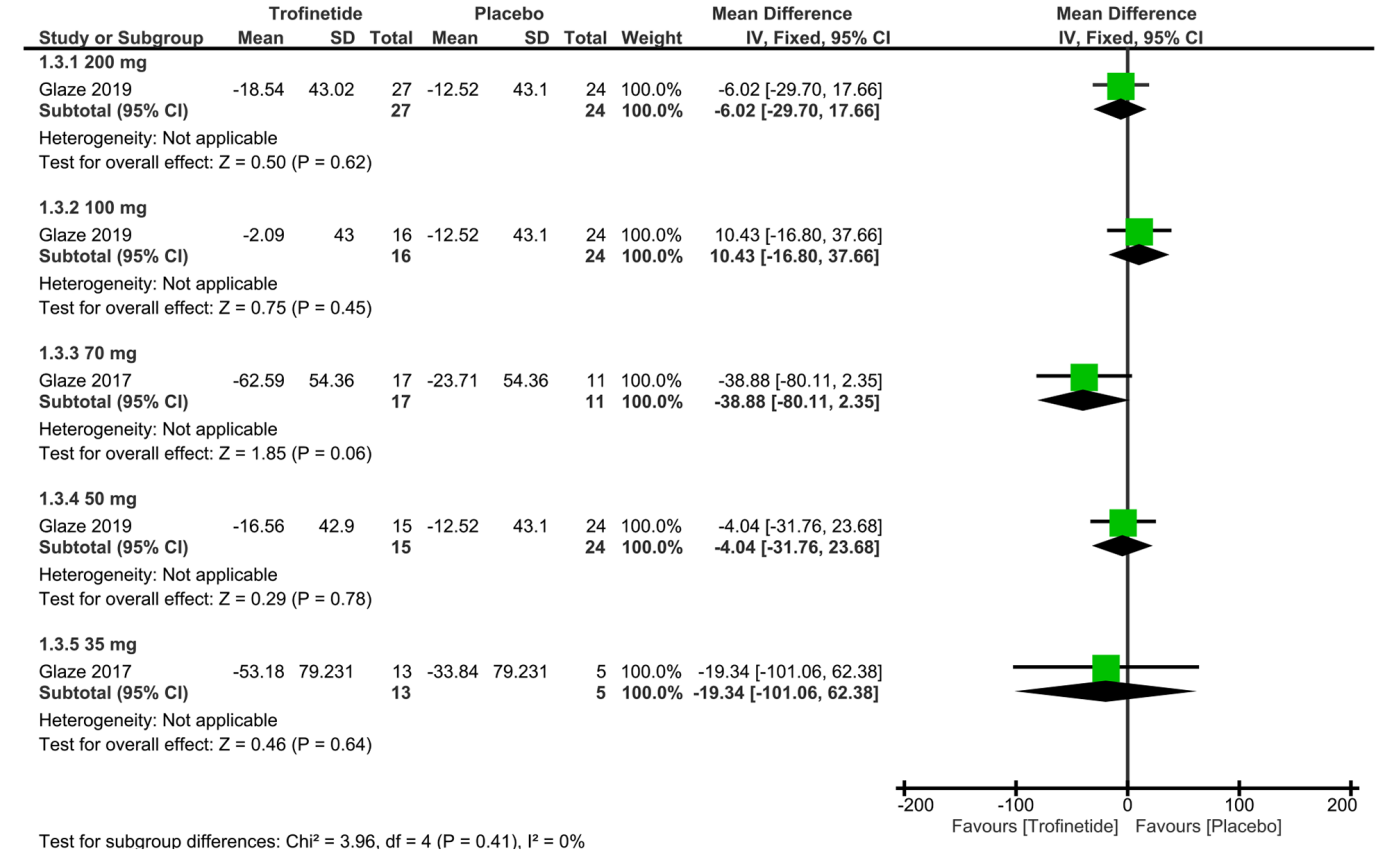
Shows the analysis of top 3 caregiver concerns between trofinetide and placebo groups at various dosages

Diving into the specifics of a single study focused on the 200 mg dosage [13], the overall mean difference in top 3 caregiver concerns was found to be -6.02 (95% CI: -29.70, 17.66, *p* = 0.62), demonstrating a negligible distinction between the two groups (Fig. 5).

#### Analysis at 100 mg dosage

##### RSBQ scores

One study reported RSBQ scores at the 100 mg dosage [13]. The calculated overall mean difference in RSBQ scores was 0.80 (95% CI: -4.07, 5.67, *p* = 0.75), revealing a statistically insignificant difference between the two arms (Fig. 2).

##### CGI-I scores

One study investigated CGI-I scores at the 100 mg dosage [13]. The overall mean difference in CGI-I scores was -0.10 (95% CI: -0.53, 0.33, *p* = 0.65), suggesting a statistically insignificant disparity between the two groups (Fig. 3).

##### MBA scores

A single study evaluated MBA scores at the 100 mg dosage. The resultant overall mean difference in MBA scores was 0.20 (95% CI: -2.89, 3.29, *p* = 0.90), indicating an insignificant difference between the two groups (Fig. 4).

##### Top 3 caregiver concerns

Based on one study assessing top 3 caregiver concerns at the 100 mg dosage [13], the overall mean difference was 10.43 (95% CI: -16.80, 37.66, *p* = 0.45), implying insignificant difference between the two groups (Fig. 5).

#### Analysis at 70 mg dosage

##### CGI-I scores

Similarly, a single study provided CGI-I scores at the 70 mg dosage [14]. The overall mean difference in CGI-I scores amounted to -0.40 (95% CI: -0.95, 0.15, *p* = 0.15), demonstrating a statistically insignificant difference between the trofinetide group and the placebo group (Fig. 3).

##### MBA scores

Similarly, one study reported MBA scores at the 70 mg dosage [14]. The overall mean difference in MBA scores was -1.39 (95% CI: -3.21, 0.43, *p* = 0.13), revealing a statistically insignificant difference between the two arms (Fig. 4).

##### Top 3 caregiver concerns

From the data provided by a single study on top 3 caregiver concerns at the 70 mg dosage [14], the overall mean difference was -38.88 (95% CI: -80.11, 2.35, *p* = 0.06), demonstrating a negligible distinction between the two groups (Fig. 5).

#### Analysis at 50 mg dosage

##### RSBQ scores

Likewise, within the realm of the 50 mg dosage, a single study provided insights on RSBQ scores [13]. The overall mean difference in RSBQ scores at this dosage was -0.70 (95% CI: -5.56, 4.16, *p* = 0.78), indicating a statistically insignificant difference between the two groups (Fig. 2).

##### CGI-I scores

At the 50 mg dosage, one study assessed CGI-I scores [13]. The calculated overall mean difference in CGI-I scores was -0.20 (95% CI: -0.63, 0.23, *p* = 0.36), indicating an insignificant statistical difference between the two groups (Fig. 3).

##### MBA scores

Continuing our investigation, we also examined MBA scores at the 50 mg dosage [13]. The overall mean difference in MBA scores was -0.20 (95% CI: -3.32, 2.92, *p* = 0.90), revealing insignificant statistical distinction between the two arms (Fig. 4).

##### Top 3 caregiver concerns

Data on top 3 caregiver concerns at the 50 mg dosage was reported by a single study [13]. The overall mean difference in the top 3 caregiver concerns was -4.04 (95% CI: -31.76, 23.68, *p* = 0.78), showing insignificant difference between the two groups (Fig. 5).

#### Analysis at 35 mg dosage

##### CGI-I scores

Based on data from one study [14], the overall mean difference in CGI-I scores at the 35 mg dosage was found to be -0.42 (95% CI: -1.24, 0.40, *p* = 0.31), revealing a statistically insignificant difference between the two arms (Fig. 3).

##### MBA scores

MBA scores at the 35 mg dosage were reported by one study [14]. The overall mean difference in MBA scores was 0.08 (95% CI: -2.91, 3.07, *p* = 0.96), demonstrating a statistically insignificant difference between the trofinetide group and the placebo group (Fig. 4).

##### Top 3 caregiver concerns

One study assessed top 3 caregiver concerns at the 35 mg dosage [14]. The overall mean difference was calculated to be -19.34 (95% CI: -101.06, 62.38, *p* = 0.64), unveiling a statistically insignificant disparity between the two groups (Fig. 5).

#### Overall analysis of TEAEs at various dosages

##### Evaluation of diarrhea odds ratio (OR)

We conducted a comprehensive analysis of diarrhea OR across the three included studies [6, 13, 14].

At the 200 mg dosage, two studies [6, 13] yielded an overall OR of 18.93 (95% CI: 9.49, 37.74, *p* < 0.00001), indicating a statistically significant distinction in diarrhea occurrence between the trofinetide and placebo groups, favoring placebo (Fig. S2).

At the 35 mg, 50 mg, 70 mg, and 100 mg dosages, individual studies consistently revealed non-significant differences in diarrhea occurrence between the trofinetide and placebo groups (Fig. S2).

##### Evaluation of vomiting OR

Our comprehensive examination extended to vomiting OR across the three included studies [6, 13, 14].

At the 200 mg dosage, the combined OR from two studies [6, 13] was 3.07 (95% CI: 1.49, 6.32, *p* = 0.002), indicating significantly increased occurrence of vomiting in the trofinetide group (Fig. S3).

Data from individual studies consistently indicated insignificant differences in vomiting occurrence between the two groups at the 35 mg, 50 mg, 70 mg, and 100 mg dosages (Fig. S3).

##### Evaluation of pyrexia OR

Our analysis encompassed the pyrexia OR across the three studies [6, 13, 14].

At the 200 mg dosage, the combined OR from two studies [6, 13] was 1.32 (95% CI: 0.46, 3.78, *p* = 0.60), revealing a lack of statistically significant difference in pyrexia occurrence between the trofinetide and placebo groups (Fig. S4).

On the other hand, studies involving the 50 mg, 70 mg, and 100 mg dosages demonstrated non-significant differences in pyrexia occurrence between the two groups (Fig. S4).

##### Evaluation of irritability OR

We further analyzed the irritability OR across the three studies [6, 13, 14].

At the 200 mg dosage, the combined OR from two studies [6, 13] was 8.19 (95% CI: 1.02, 65.80, *p* = 0.05), denoting a statistically significant increase in irritability occurrence in the trofinetide group (Fig. S5).

Studies at the 35 mg, 70 mg, and 100 mg dosages yielded non-significant differences in irritability occurrence between the two groups (Fig. S5).

##### Evaluation of decreased appetite OR

Lastly, our analysis encompassed decreased appetite OR across the three included studies [6, 13, 14].

At the 200 mg dosage, the combined OR from two studies [6, 13] was found to be 1.56 (95% CI: 0.40, 6.04, *p* = 0.52), indicating no significant difference in the occurrence of decreased appetite between the trofinetide group and the placebo group (Fig. S6).

In the study by Glaze et al. (2019), analysis of the 100 mg and 50 mg dosages revealed non-significant differences in the incidence of decreased appetite between the two groups (Fig. S6).

## Discussion

Our findings regarding the efficacy of trofinetide in Rett syndrome patients showed that using a 200 mg/kg bid of trofinetide is associated with better outcomes regarding RSBQ and CGI-I compared to placebo. However, there was no significant difference between trofinetide and placebo in both MBA and the caregiver top 3 concerns Visual Analog Scale (VAS) results. Moreover, lower doses such as 100 mg, 70 mg, 50 mg, and 35 mg had the same significance in the outcomes as placebo. For safety outcomes, the 200 mg/kg bid dose of trofinetide was associated with an increased risk of diarrhea and vomiting compared with the placebo with an OR of 18.93 (95% CI: 9.49, 37.74) and 3.07 (95% CI: 1.49, 6.32), respectively. However, using lower doses did not lead to a highly significant OR regarding the risk of all side effects, including diarrhea and vomiting. Furthermore, a 200 mg/kg bid of trofinetide showed no association with an increased risk of irritability, decreased appetite, or pyrexia. The summary of TEAEs at each dose in the trofinetide group is illustrated in Table 3.

**Table 3:** Shows the summary of the trofinetide Treatment Emergent Adverse Events (TEAEs) at different doses

Dose, mg	Diarrhea, %	Irritability, %	Pyrexia, %	Vomiting, %	Decreased appetite, %
**200**	75.0	5.8	6.7	25.8	4.2
**100**	12.5	6.3	0.0	12.5	0.0
**70**	11.1	0.0	11.1	11.1	-
**50**	26.7	-	0.0	6.7	6.7
**35**	38.9	22.2	-	0.0	-

Due in part to its disorder-specificity and reliability and validity, particularly for the Rett syndrome pediatric population, the RSBQ is the most commonly used behavioral instrument in Rett syndrome [15–18]. The RSBQ has recently demonstrated increased sensitivity to interventions and relationships with functioning and quality of life in Rett syndrome [17, 19, 20]. Given that the Rett syndrome elements in the RSBQ are modulated rather than triggered by behavior (such as breathing issues), the measure might be better described as “neurobehavioral” in this sense. Consequently, the RSBQ is a tool similar to the MBA that may be used to evaluate various basic Rett syndrome properties [13]. However, our study showed no efficacy of trofinetide on MBA, and this can be attributed to the small sample size due to a low number of included studies that failed to reach statistical significance.

The efficacy parameters are matched and indicate functionally significant aspects of Rett syndrome, such as communication ability. The RSBQ has associations with functioning. It is validated across ages (2-47 years) in Rett syndrome [16–18]. The CGI-I scale has been frequently utilized in clinical trials for Rett syndrome and other neurological disorders. It is a clinician rating and gives clinical relevance to the caregiver-rated coprimary objective [6, 13, 14, 21–24]. This shows the significance of our findings since we observed the efficacy of trofinetide in improving these scales in patients with Rett syndrome compared with placebo.

Glaze and colleagues [13] conducted a phase 2 trial in 2019 on pediatric and adolescent patients to examine the efficacy and safety of trofinetide in Rett syndrome. This study showed high efficacy regarding RSBQ and the clinician Domain Specific Concerns-Visual Analog Scale (DSC-VAS). These findings are consistent with the results of the CGI-I functional improvement in the same study. Significant improvements were seen in various symptom categories and specific symptoms, including repetitive behaviors, breathing issues, mood abnormalities/disruptive behavior, ambulation difficulties, and seizures, according to the RSBQ and DSC-VAS data [13]. These findings go along with the results of Glaze et al. in their trial conducted in 2017 [14] On adults and adolescents, which demonstrated improvement in measures addressing various illness symptoms (such as MBA and CGI-I). This also comes in agreement with the findings from experimental studies concerning the overall mechanism of action of trofinetide [25, 26].

Regarding the used dose, we found that the 200 mg/kg bid dose of trofinetide is the only effective used dose in Rett syndrome patients regarding RSBQ and CGI-I. Glaze and Colleagues in 2019 [13] raised the used dose to 50 mg, 100 mg, and 200 mg/kg bid compared to 35 mg and 70 mg as used in the study conducted in 2017. They found that clinical improvement was observed only in the highest dose as well as the longer treatment duration applied in the second study (42 days) compared with the first study (28 days) [13, 14].

Although the highest-used dose of trofinetide (200 mg/kg bid) was the only effective dose as present by the included studies, it was the only dose associated with increased side effects, mainly diarrhea and vomiting. In Neul’s study [6], diarrhea was reported with 81% of patients taking 200 mg/kg bid of trofinetide. This study also showed that most trofinetide discontinuations were due to at least one TEAE, most of which were mild or moderate diarrhea; nevertheless, the diarrhea was self-limited and went away quickly following trofinetide withdrawal. The participants who received trofinetide and completed the study was 75% [6]. This success can be attributed to the implementation of a diarrhea-management plan halfway through the study; the plan involved adjustment or discontinuation of laxative medications frequently taken for Rett syndrome-associated constipation [6]. The plan also included initiation of fiber supplements and antidiarrheal medicines and reducing or temporarily stopping the use of trofinetide if necessary. Overall, this approach seemed to reduce the risk of diarrhea [6]. In addition, Glaze et al. [13] also reported that 56% of the patients taking 200 mg/kg bid suffered from diarrhea, and 22% suffered from vomiting. These side effects were self-limited after discontinuing the drug; none affected the tolerability.

In this systematic review and meta-analysis, we pooled the results of all available RCTs investigating trofinetide in Rett syndrome, a debilitating neurodevelopmental condition for which no pharmacotherapies directed at core features are available. We included all the known efficacy and safety outcomes to assess its application in the clinical field comprehensively. In addition, we investigated the efficacy and safety of different doses to guide clinicians toward the best practice.

On the other hand, there exist some limitations in our study. This is mainly represented in the few studies currently published in the literature investigating our idea. This, in turn, leads to a small sample size in the pooled analysis, which may produce insignificant results. Moreover, two studies [6, 13] used the same dose, 200 mg/kg bid, and showed its efficacy. However, the other lower doses were only presented in one study for each [13, 14]. This does not allow a final decision toward these doses. Furthermore, two studies [6, 13] were conducted on children and adolescents and one study [14] was conducted on adults and adolescents. Therefore, further multicenter RCTs with a large sample size must be performed. These RCTs should compare patients using different doses of the drug and should incorporate other age groups of the population.

## Conclusion

In conclusion, our study provides valuable insights regarding the safety and efficacy of trofinetide in Rett syndrome. Our comprehensive analysis across different dosages revealed distinct patterns of response and adverse events. The significant improvement observed in RSBQ and CGI-I scores at the 200 mg dosage highlights the therapeutic potential of trofinetide and the importance of dosage considerations.

The nuanced relationship between trofinetide dosages and efficacy outcomes, as demonstrated by insignificant changes in MBA and top 3 caregiver concerns, emphasizes the need for personalized treatment strategies. Moreover, the association between specific dosages and the occurrence of adverse events underscores the delicate balance between efficacy and tolerability.

### Electronic supplementary material

Below is the link to the electronic supplementary material.

Supplementary Material 1

## Data Availability

This systematic review and meta-analysis relied on publicly available data from previously published studies. The original research contributions utilized in this study can be accessed within the main article and supplementary materials.

## Abbreviations

RSBQ: Rett Syndrome Behavior Questionnaire
CGI-I: Clinical Global Impression-Improvement
MBA: Motor Behavioral Assessment
RCTs: Randomized Controlled Trials
PRISMA: Preferred Reporting Items for Systematic Reviews and Meta-Analyses
WoS: Web of Science
OR: Odds Ratio
CNS: Central Nervous System
ROB: Risk of Bias
ASD: Autism Spectrum Disorders
CI: Confidence Interval
p: p-value
