# Supplementary material for: Safety and efficacy of trofinetide in Rett syndrome: a systematic review and meta-analysis of randomized controlled trials
Source: BMC Pediatr. 2024 Mar 23;24:206. doi: 10.1186/s12887-024-04526-3 (PMC10960414; doi:10.1186/s12887-024-04526-3)
Supplement: Supplementary file 1 — Supplementary Material 1 [file 12887_2024_4526_MOESM1_ESM.docx]

**Fig. S1. Quality assessment by RoB.2 tool**

**
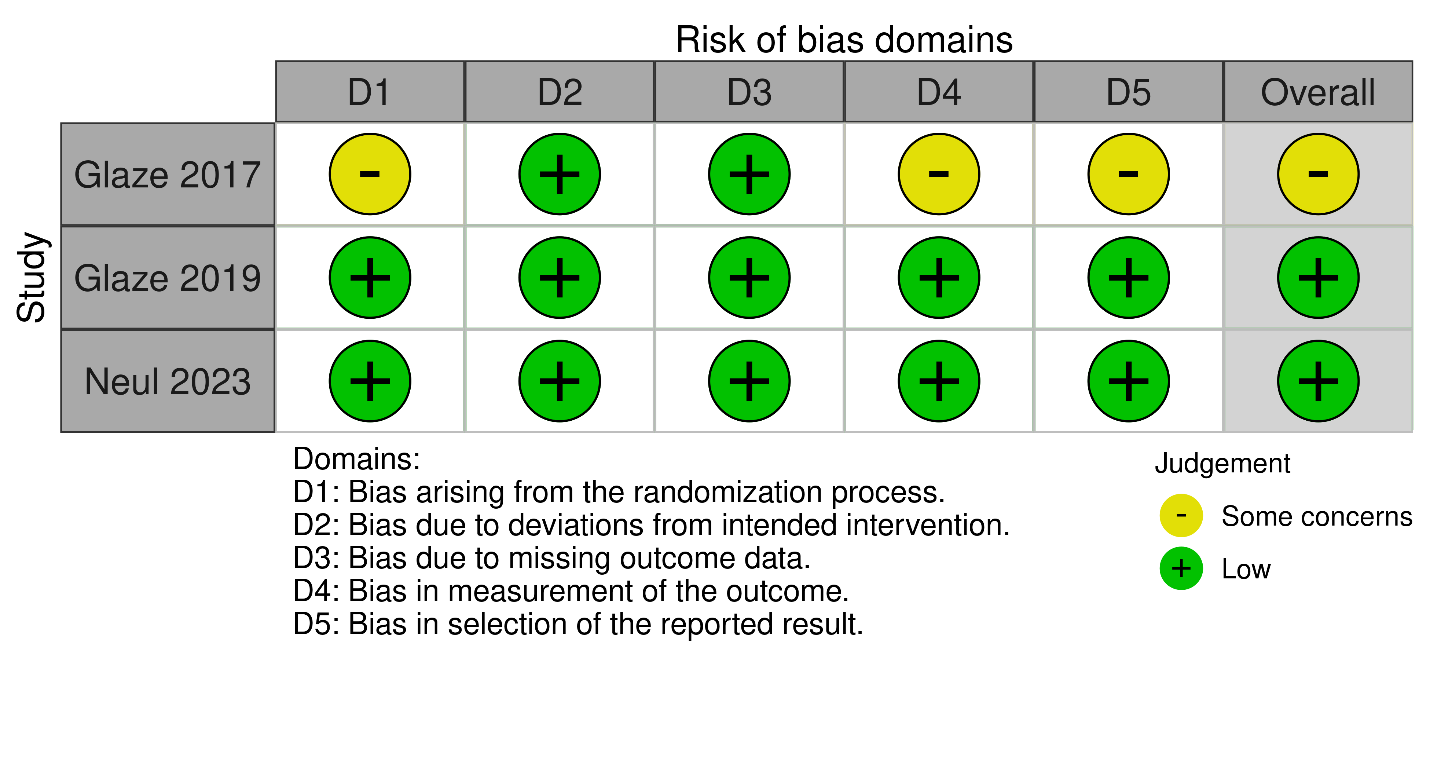
**

**Fig. S2. The analysis of the diarrhea between the trofinetide and placebo groups at various dosages**

**
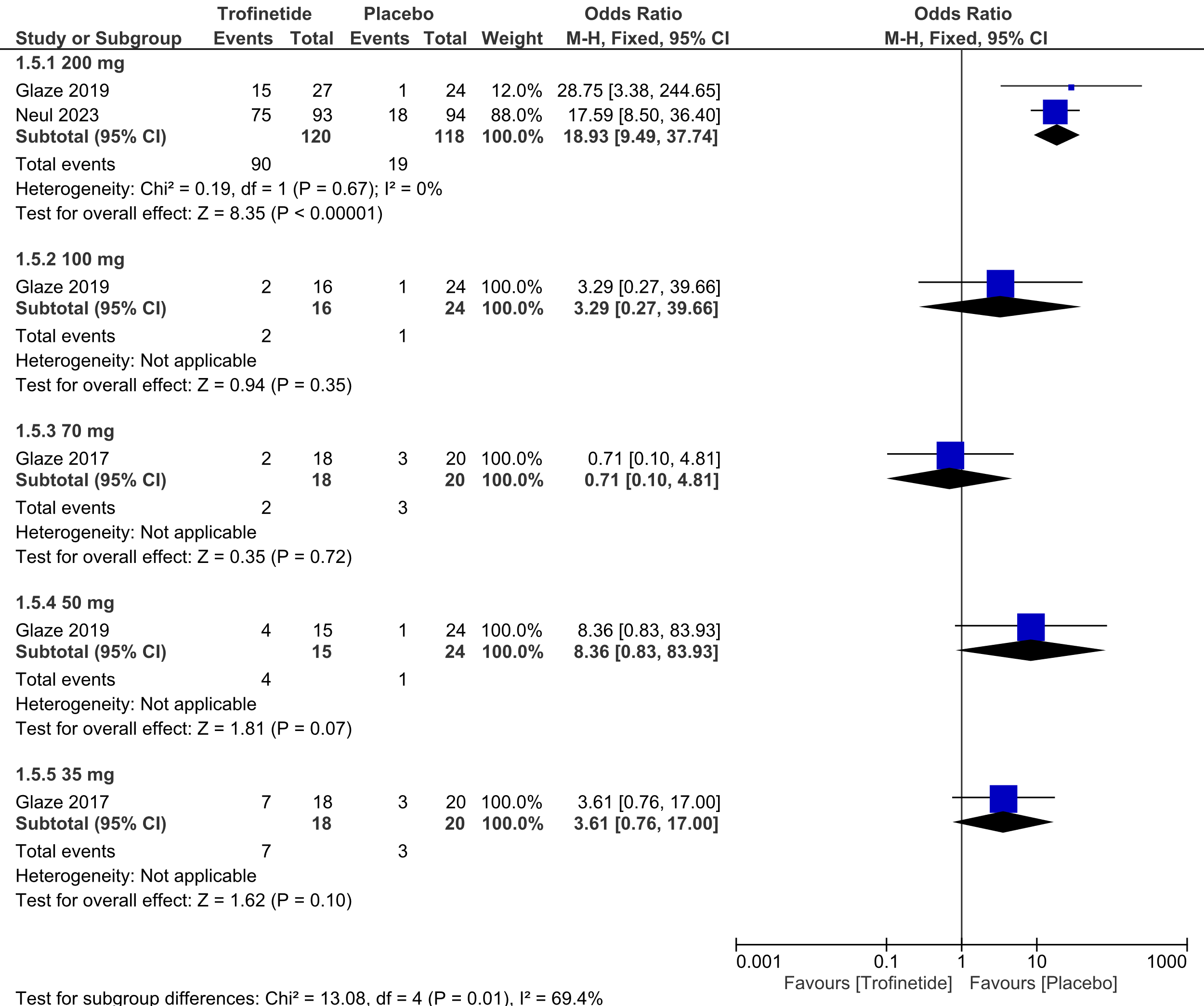
**

**Fig. S3. The analysis of the vomiting between the trofinetide and placebo groups at various dosages**

**
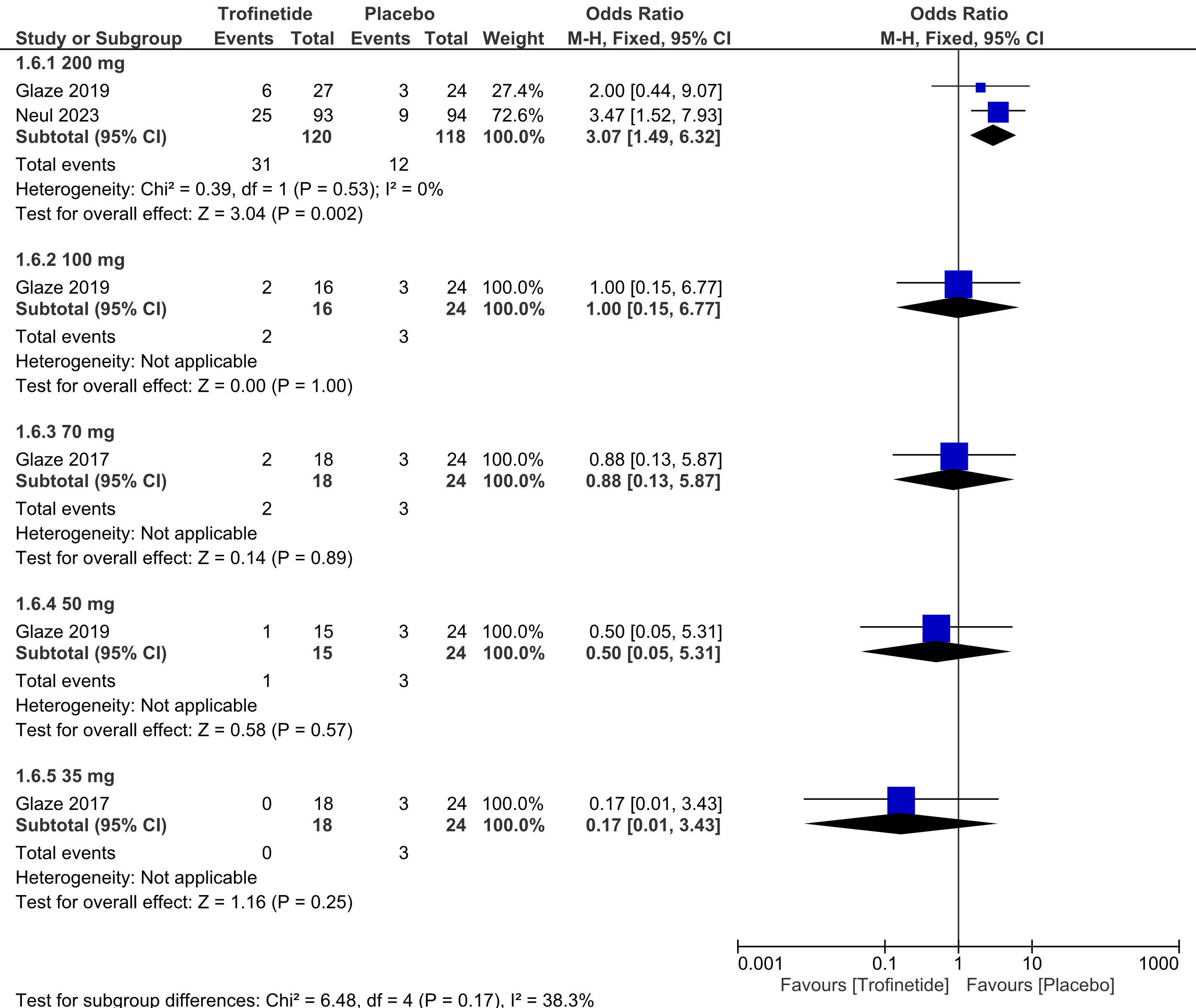
**

**Fig. S4. The analysis of the pyrexia between the trofinetide and placebo groups at various dosages**

**
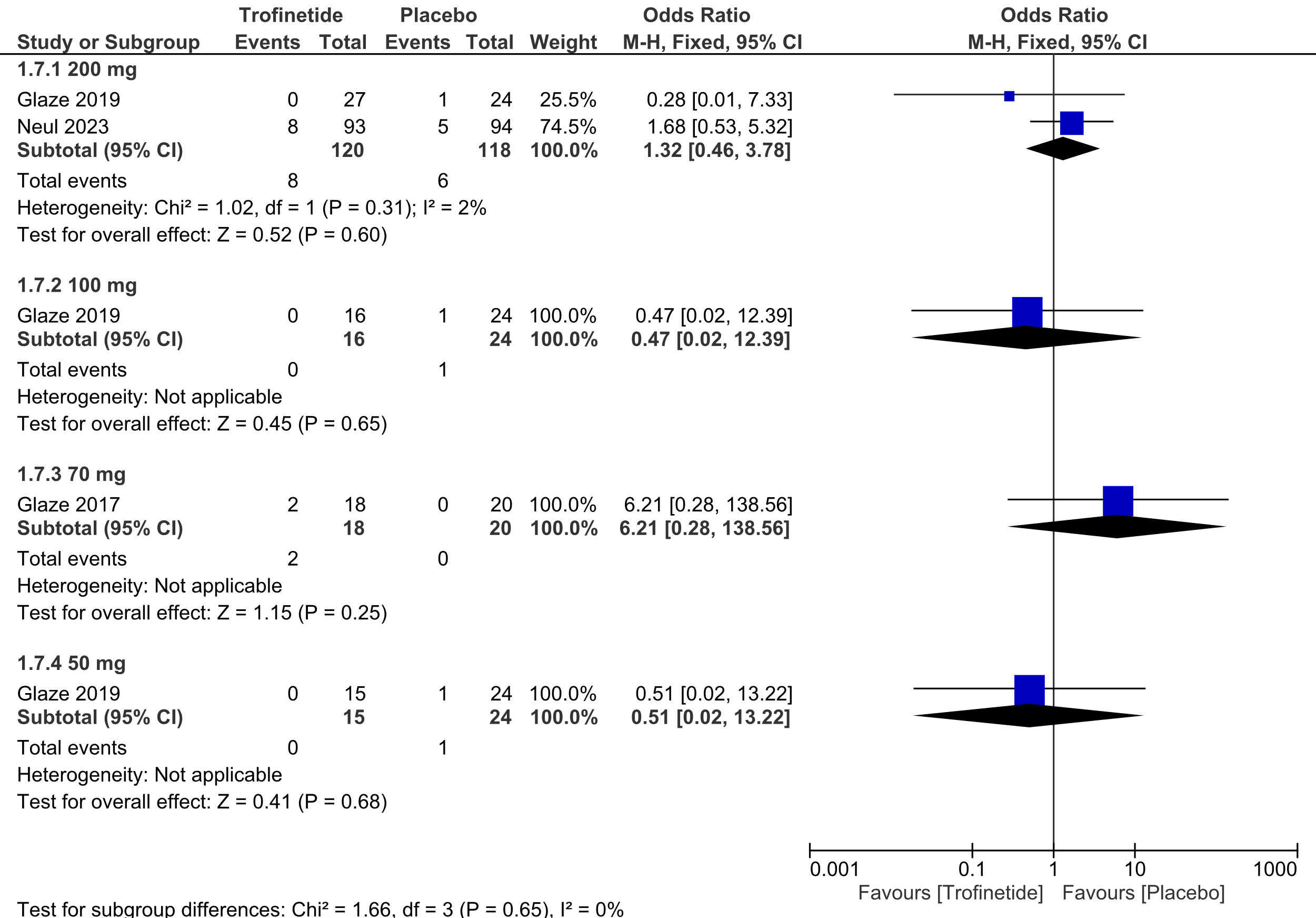
**

**Fig. S5. The analysis of the irritability between the trofinetide and placebo groups at various dosages**

**
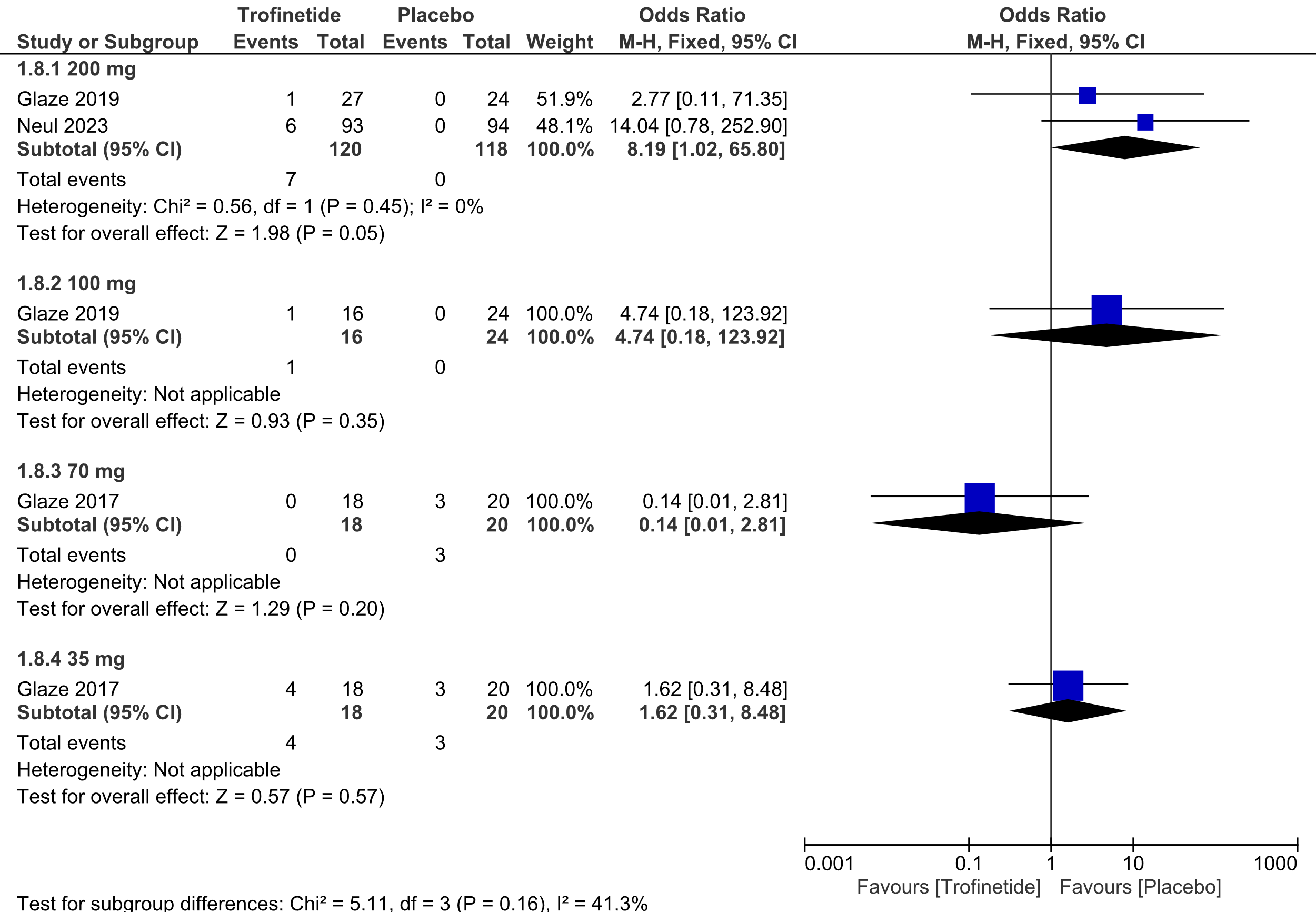
**

**Fig. S6. The analysis of the decreased appetite between the trofinetide and placebo groups at various dosages**

**
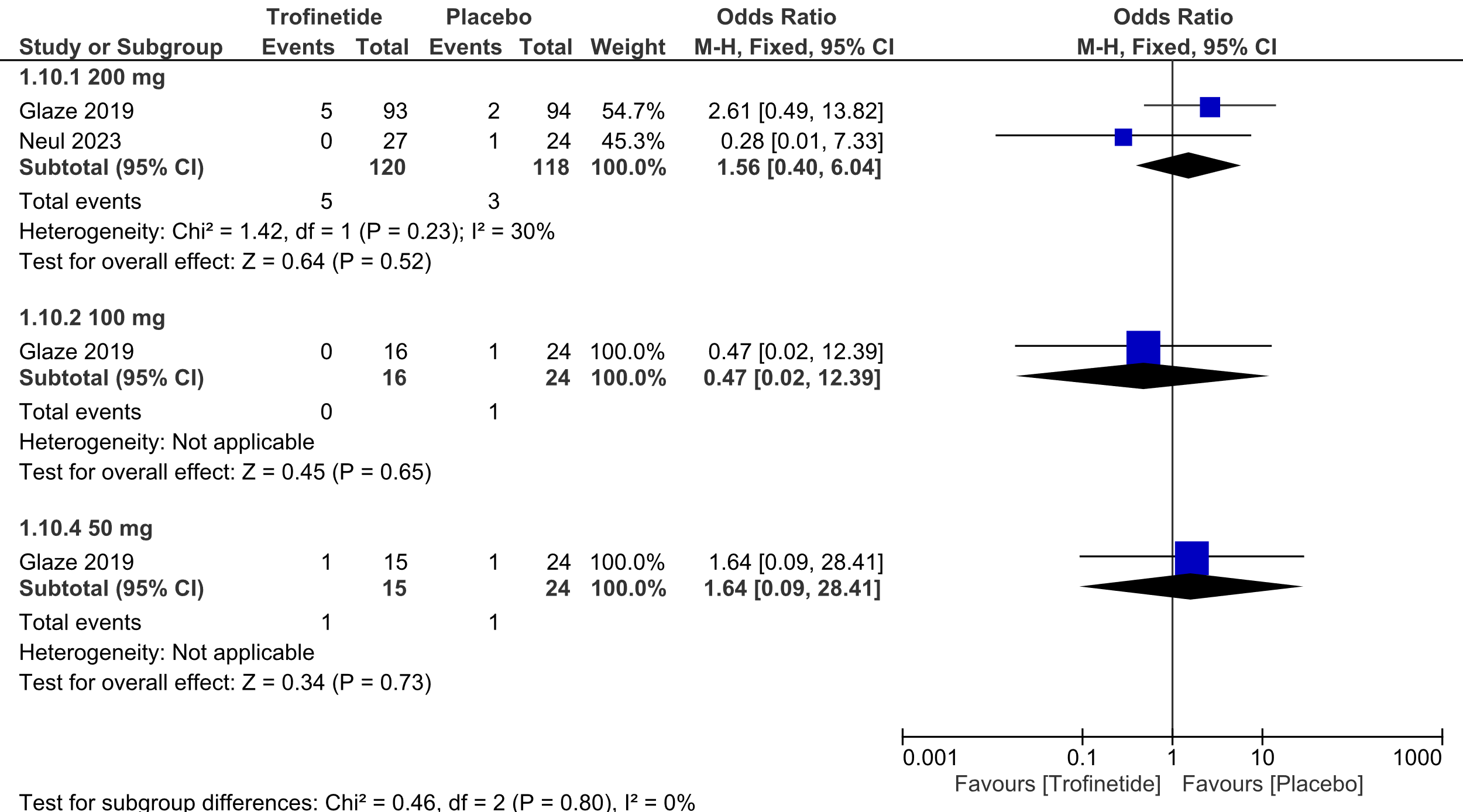
**
